# Treatment patterns and survival outcomes of advanced hypopharyngeal squamous cell carcinoma

**DOI:** 10.1186/s12957-020-01866-z

**Published:** 2020-05-01

**Authors:** Yao-Te Tsai, Wen-Cheng Chen, Chih-Yen Chien, Cheng-Ming Hsu, Yi-Chan Lee, Ming-Shao Tsai, Meng-Hung Lin, Chia-Hsuan Lai, Kai-Ping Chang

**Affiliations:** 1 Department of Otorhinolaryngology-Head and Neck Surgery, Chiayi Chang Gung Memorial Hospital, Chiayi, Taiwan; 2 Department of Otolaryngology–Head and Neck Surgery, Linkou Chang Gung Memorial Hospital, No. 5 Fu-Hsing street, Taoyuan, Taiwan 33305; 3 College of Medicine, Chang Gung University, Taoyuan, Taiwan; 4 Department of Radiation Oncology, Chiayi Chang Gung Memorial Hospital, Chiayi, Taiwan; 5 Department of Otorhinolaryngology-Head and Neck Surgery, Kaohsiung Chang Gung Memorial Hospital, Kaohsiung, Taiwan; 6 Department of Otorhinolaryngology-Head and Neck Surgery, Keelung Chang Gung Memorial Hospital, Keelung, Taiwan; 7 Health Information and Epidemiology Laboratory, Chiayi Chang Gung Memorial Hospital, Chiayi, Taiwan

**Keywords:** Head and neck, Cancer, Hypopharynx, Squamous cell carcinoma, Prognosis

## Abstract

**Background:**

This study evaluated the treatment outcomes of the primary surgery (PS) or concurrent chemoradiotherapy (CCRT) as the initial treatment for hypopharyngeal squamous cell carcinoma (HPSCC).

**Methods:**

This retrospective cohort study included patients with stages III–IV HPSCC from four tertiary referral centers consecutively enrolled from 2003 to 2012; of them, 213 (32.6%) and 439 (67.4%) had received PS and CCRT as their primary treatments, respectively. The 5-year overall survival (OS) and disease-free survival (DFS) rates were analyzed using the Kaplan–Meier method and Cox regression models.

**Results:**

In patients undergoing PS and CCRT, OS rates were 45.0% and 33.1% (*p* < 0.001), respectively, and DFS rates were 36.2% and 28.9% (*p* = 0.003), respectively. In subgroup analysis, in patients with stage IVA HPSCC, PS was associated with higher OS rate (*p* = 0.002), particularly in those with T4 or N2 classification (*p* = 0.021 and 0.002, respectively). Multivariate analysis indicated that stage IVA HPSCC, stage IVB HPSCC, and CCRT were independent adverse prognostic factors for OS rate (*p* = 0.004, < 0.001, and 0.014, respectively). Furthermore, in patients with stage IVA HPSCC aged ≥ 65 years and with N2 classification, CCRT was significantly associated with lower OS rates than was PS (*p* = 0.027 and 0.010, respectively).

**Conclusions:**

In patients with advanced HPSCC, PS was significantly associated with better prognosis than CCRT. PS could be a favorable primary treatment modality for the management of patients with stage IVA HPSCC, particularly those aged ≥ 65 years and with T4 and N2 classification.

## Introduction

Hypopharyngeal squamous cell carcinoma (HPSCC) accounts for 3–5% of all head and neck cancers, and approximately 60–85% of patients with HPSCC patients have stages III–IV disease at the time of diagnosis [1–3] and thus demonstrate poor prognosis irrespective of contemporary aggressive multidisciplinary treatments [4]. Delayed diagnosis due to the lack of initial symptoms, the propensity of submucosal spread, the high rate of clinically positive nodes at presentation, and the high incidences of recurrence and second primary tumors may also contribute poor prognosis [5–7]. Because of the anatomic proximity of the larynx, and the desire to preserve respiratory, deglutition, and speech functions, additional consideration when choosing treatment modalities for patients with HPSCC is warranted. Before the 1990s, radical surgery with total or partial laryngectomy was considered a mainstay for advanced-stage HPSCC treatment, with the 5-year overall survival (OS) rate varying from 10 to 60% [3, 8, 9]. In the late 1990s, the results of retrospective and prospective studies, including the Veterans Affairs trials, European Organization for Research and Treatment of Cancer (EORTC) trial 24891 (definitive treatment), EORTC trial 22931, and Radiation Therapy Oncology Group (RTOG) trial 9501 (adjuvant), began the trend of nonsurgical treatments involving radiotherapy combined with platinum-based chemotherapy as favorable alternatives to surgery to preserve the larynx in patients with resectable advanced-stage HPSCCs [10–13]. However, despite the wide acceptance of concurrent chemoradiotherapy (CCRT) as a primary treatment modality for HPSCC, recent evidence suggests that radical primary surgery (PS) may provide superior survival outcome in patients with HPSCC [14, 15]. The optimal initial treatment for patients with advanced-stage HPSCC remains under debate [16].

The current multicenter retrospective study was conducted to present the oncological results of PS with and without adjuvant therapy and definitive CCRT as the initial primary treatment modality and illustrate which approach could be the optimal initial treatment among subgroups (particularly T or N classification) of advanced-staged HPSCC. The primary endpoints were the long-term survival outcomes of patients with advanced-stage HPSCC treated with either definitive CCRT or PS followed by adjuvant therapy. We also identified the subgroup of patients with advanced-stage HPSCC with the highest survival rate after treatment with PS or CCRT.

## Materials and methods

### Data source

This study extracted data of patients with advanced-stage HPSCC from four major hospitals within the health care system from the Cancer Registry and Death Registration of Chang Gung Medical Foundation in Taiwan. This database provides complete and high-quality information regarding the individual demographics, clinical diagnosis codes, cancer stages, tumor histology, and primary treatment details, which have been suggested to be of high quality [17]. The study followed strict confidentiality guidelines according to the regulations on personal electronic data protection and was approved by the Institutional Review Board of Chang Gung Medical Foundation.

### Study population and design

**Fig. 1:**
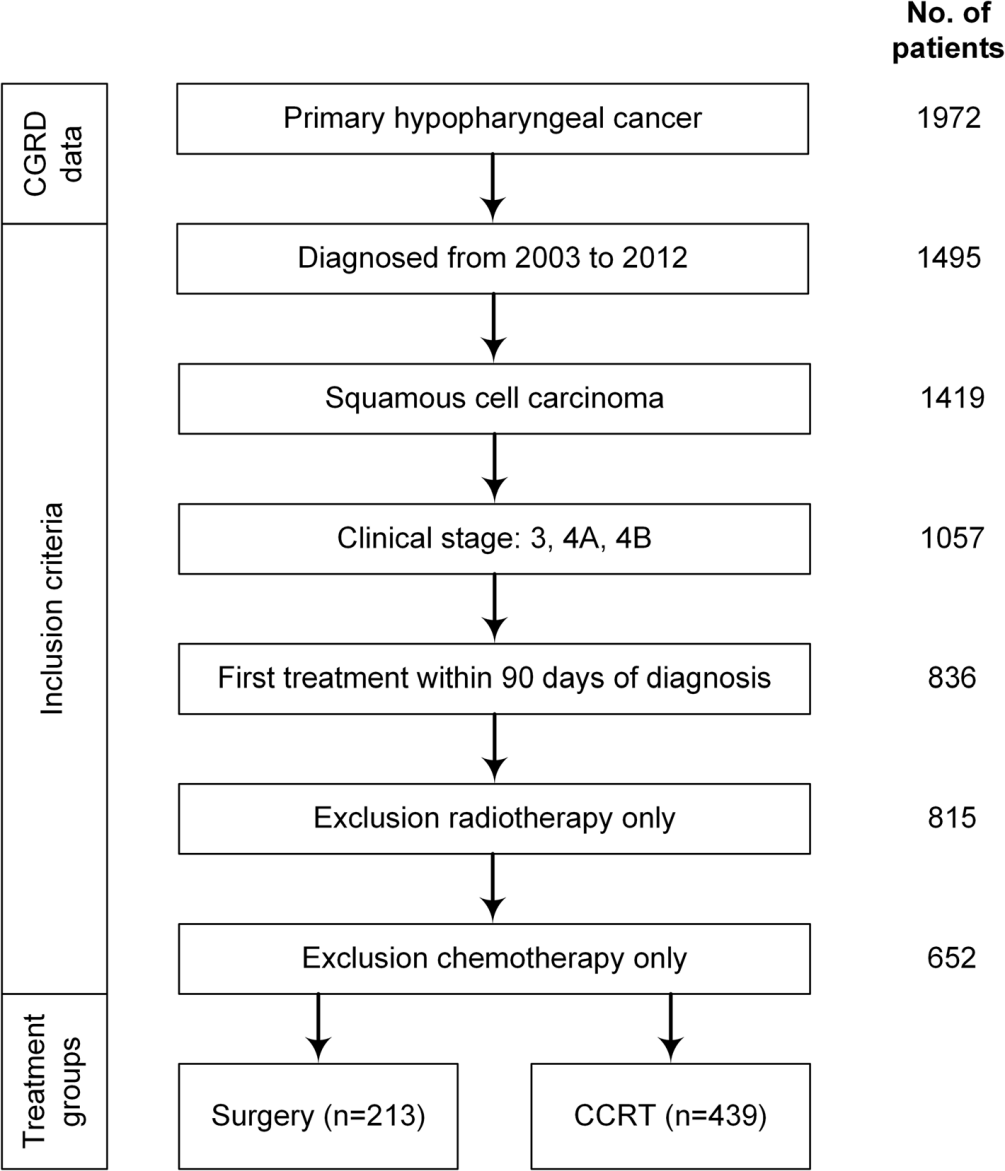
Study flow chart. Only patients with stages III–IV HPSCC were enrolled

We included all patients from the database who were newly diagnosed with advanced-stage (stages III, IVA, or IVB) HPSCC between January 1, 2003 and December 31, 2012, and all medical records of the study cohort were extracted from the database and analyzed. Demographic characteristics, including age at diagnosis, overall clinical stages (T and N classifications) based on the American Joint Committee on Cancer Staging Manual (2010), and the duration and dosage of the chemoradiotherapy, were recorded. Patients with prior cancer before the first day of HPSCC diagnosis, a synchronous second primary malignancy, distant metastasis at presentation, no cancer treatment on either arm for more than 3 months after diagnosis, treatment with chemotherapy or radiotherapy alone, incomplete CCRT course or neoadjuvant therapy (chemotherapy or radiotherapy) before surgery, and a primary tumor excised by transoral laser or robotic surgery were excluded from the study. All enrolled subjects were followed up until the end of 2015 or death. The flow chart of the current study is depicted in Fig. 1.

Outcome endpoints for survival analyses were 5-year overall survival (OS) and disease-free survival (DFS) rates. Patients treated with PS underwent total or partial laryngopharyngectomy with or without regional or free-flap tissue transfer based on the disease extent and physicians’ preferences, and all patients received unilateral or bilateral neck dissection simultaneously. If definitive CCRT was selected as the initial treatment, the radiotherapy dose was at least 70 Gy.

### Statistical analysis

Kaplan–Meier plot was used to illustrate the 5-year OS and DFS rates, and the log-rank method was used for univariate comparison between the survival curves. Cox proportional hazard models were adapted to measure the hazard ratios (HRs) and their 95% confidence intervals (CIs) in univariate and multivariate analyses after adjustments for the treatment modalities and clinical characteristics. A *p* of < 0.05 indicated statistical significance. All analyses were conducted by using the SAS statistical software (version 9.2; SAS Institute, Cary, NC, USA).

## Results

### Patient characteristics

Between January 1, 2003 and December 31, 2012, 1057 patients were newly diagnosed as having stage III or IV HPSCC in four individual hospitals. Patients who received the first treatment > 90 days after diagnosis (*n* = 221), underwent radiotherapy alone (*n* = 21), or received chemotherapy alone (*n* = 163) were excluded (Fig. 1). Finally, 652 patients were followed until the end of 2015 or death; of them, 213 (32.7%) and 439 (67.3%) had undergone PS and definitive CCRT as the initial treatment modality, respectively. In the PS group, 151 (70.8%) patients received postoperative adjuvant therapy, and salvage surgery was performed in 168 patients (38.3%) of the CCRT group. The characteristics of all patients are presented in Table 1.

**Table 1:** Baseline characteristics of advanced squamous cell carcinoma of the hypopharynx by primary treatment.

Variable	All		Surgery		CCRT	
	*n*	(%)	*n*	(%)	*n*	(%)
Total	652		213		439	
Sex						
Female	13	(2.0)	1	(7.7)	12	(92.3)
Male	639	(98.0)	212	(33.2)	427	(66.8)
Age (years)						
< 65	565	(86.7)	184	(32.6)	381	(67.4)
≥ 65	87	(13.3)	29	(33.3)	58	(66.7)
Mean (SD)	53	(10.0)	53	(9.6)	53	(10.3)
T classification						
T1	30	(4.6)	10	(33.3)	20	(66.7)
T2	81	(12.4)	23	(28.4)	58	(71.6)
T3	146	(22.4)	63	(43.2)	83	(56.8)
T4	395	(60.6)	117	(29.6)	278	(70.4)
N classification						
N0	100	(15.3)	30	(30.0)	70	(70.0)
N1	99	(15.2)	48	(48.5)	51	(51.5)
N2	369	(56.6)	127	(34.4)	242	(65.6)
N3	84	(12.9)	8	(9.5)	76	(90.5)
Overall stages						
III	91	(14.0)	44	(48.4)	47	(51.6)
IVA	412	(63.2)	147	(35.7)	265	(64.3)
IVB	149	(22.8)	22	(14.8)	127	(85.2)
Histology grade						
WD/MD SCC	360	(55.2)	177	(49.2)	183	(50.8)
PD/UD	90	(13.8)	27	(30.0)	63	(70.0)
Unclassified	202	(31.0)	9	(4.5)	193	(95.5)

*CCRT* concurrent chemoradiotherapy, *WD* well-differentiated squamous cell carcinoma, *MD* moderately differentiated squamous cell carcinoma, *PD* poorly differentiated squamous cell carcinoma, *UD* undifferentiated carcinoma

### Survival analysis in the advanced-stage HPSCC

**Fig. 2:**
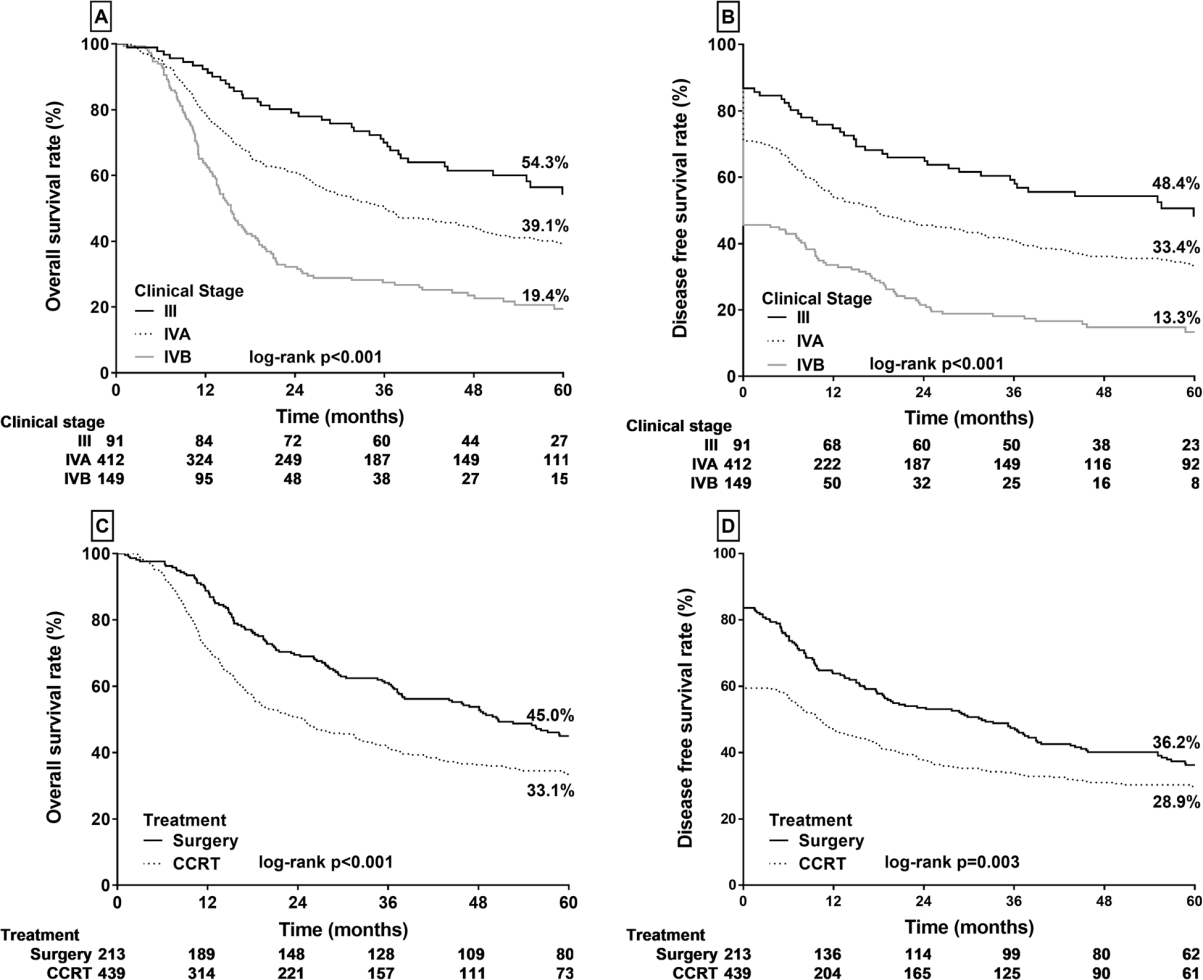
Kaplan–Meier analysis of OS and DFS rates in patients with advanced hypopharyngeal squamous cell carcinoma. **a** and **b** Stages III, IVA, and IVB. **c** and **d** PS versus CCRT

In all patients, the median follow-up duration was 30.6 months, and their 5-year OS and DFS rates were 37.5% and 31.3%, respectively. Clinical stage was a significant predictor of prognosis (Fig. 2). Kaplan–Meier survival analysis indicated that the OS rates in patients with stages III, IVA, and IVB HPSCC were 54.3%, 39.1%, and 19.4%, respectively, and their DFS rates were 48.4%, 33.4%, and 13.3%, respectively (both log-rank *p* < 0.001; Fig. 2 a and b, respectively).

In patients treated with PS and definitive CCRT, the median survival duration was 50.6 (95% CI 37.9–66.3) and 24.3 (95% CI 18.4–28.7) months, respectively. The Kaplan–Meier estimates of the OS rates in patients treated with PS and definitive CCRT were 45.0% and 33.1% (both *p* < 0.001), respectively (Fig. 2c), whereas their DFS rates were 36.2% and 28.9% (*p* = 0.003), respectively (Fig. 2d). These results indicated that the long-term prognosis after PS was superior to that after definitive CCRT.

**Fig. 3:**
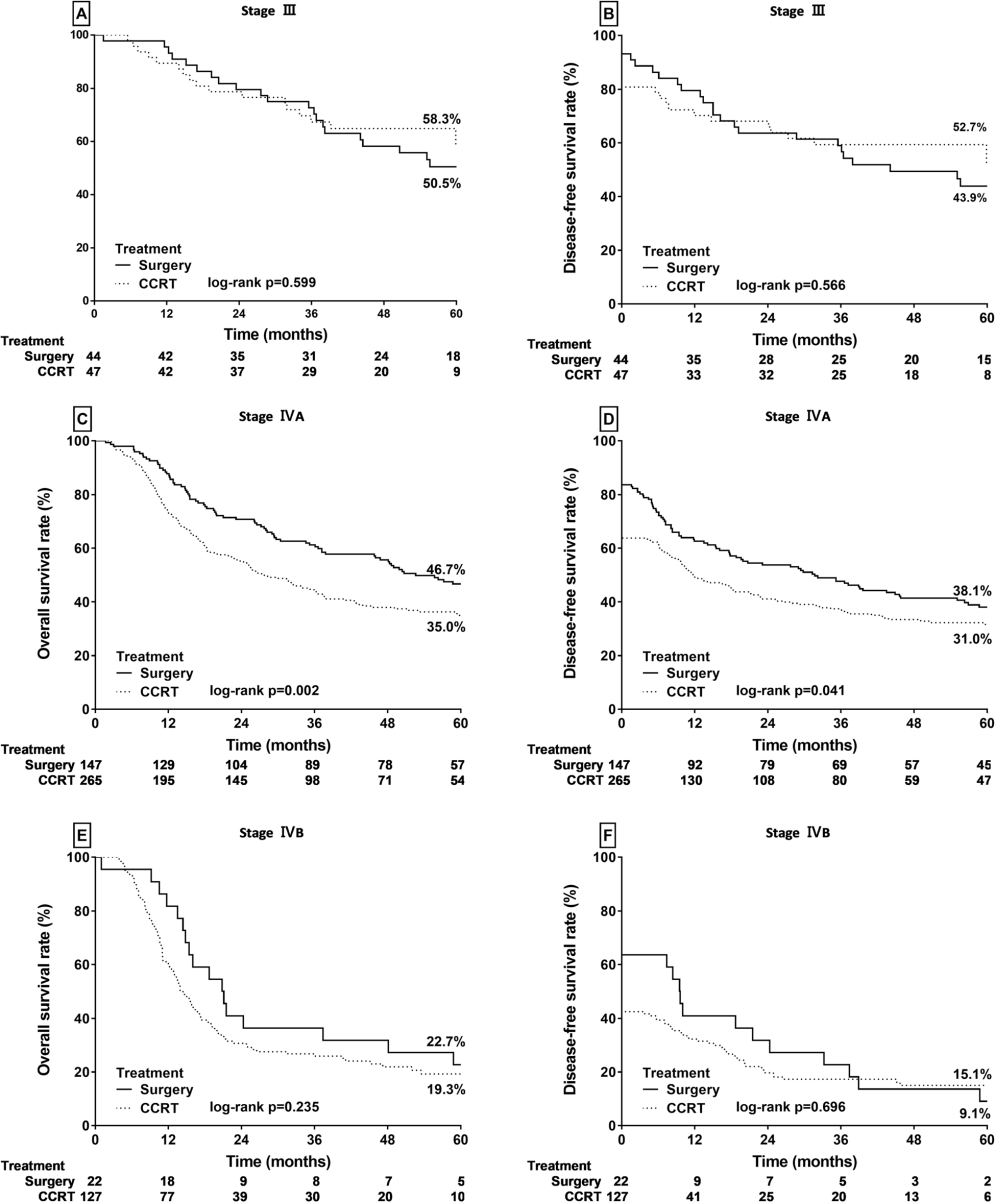
Kaplan–Meier analysis of OS and DFS rates in patients with stages III–IV HPSCC. **a** and **b** Stage III patients. **c** and **d** Stage IVA patients. **e** and **f** Stage IVB patients

We compared the survival outcome between PS and CCRT by clinical stage. In patients with stage III HPSCC, the OS and DFS rates in the PS and CCRT groups were similar but without statistical significance (Fig. 3a and b). Figure 3c and d indicate that in patients with stage IVA HPSCC, compared with definite CCRT, PS led to significantly higher OS (46.7% vs 35.0%, *p* = 0.002) and DFS (38.1% vs 31.0%, *p* = 0.041) rates. In patients with stage IVB HPSCC, OS rates were 22.7% and 19.3% after PS and definite CCRT (*p* = 0.235), respectively (Fig. 3e), and DFS rates were 15.1% and 9.1% after PS and definite CCRT (*p* = 0.696), respectively (Fig. 3f). Although patients with stage IVB HPSCC in the PS group appeared to have higher OS and DFS rates, the differences were not statistically significant.

### Subgroup survival analysis of stage IVA HPSCC by treatment modality

**Fig. 4:**
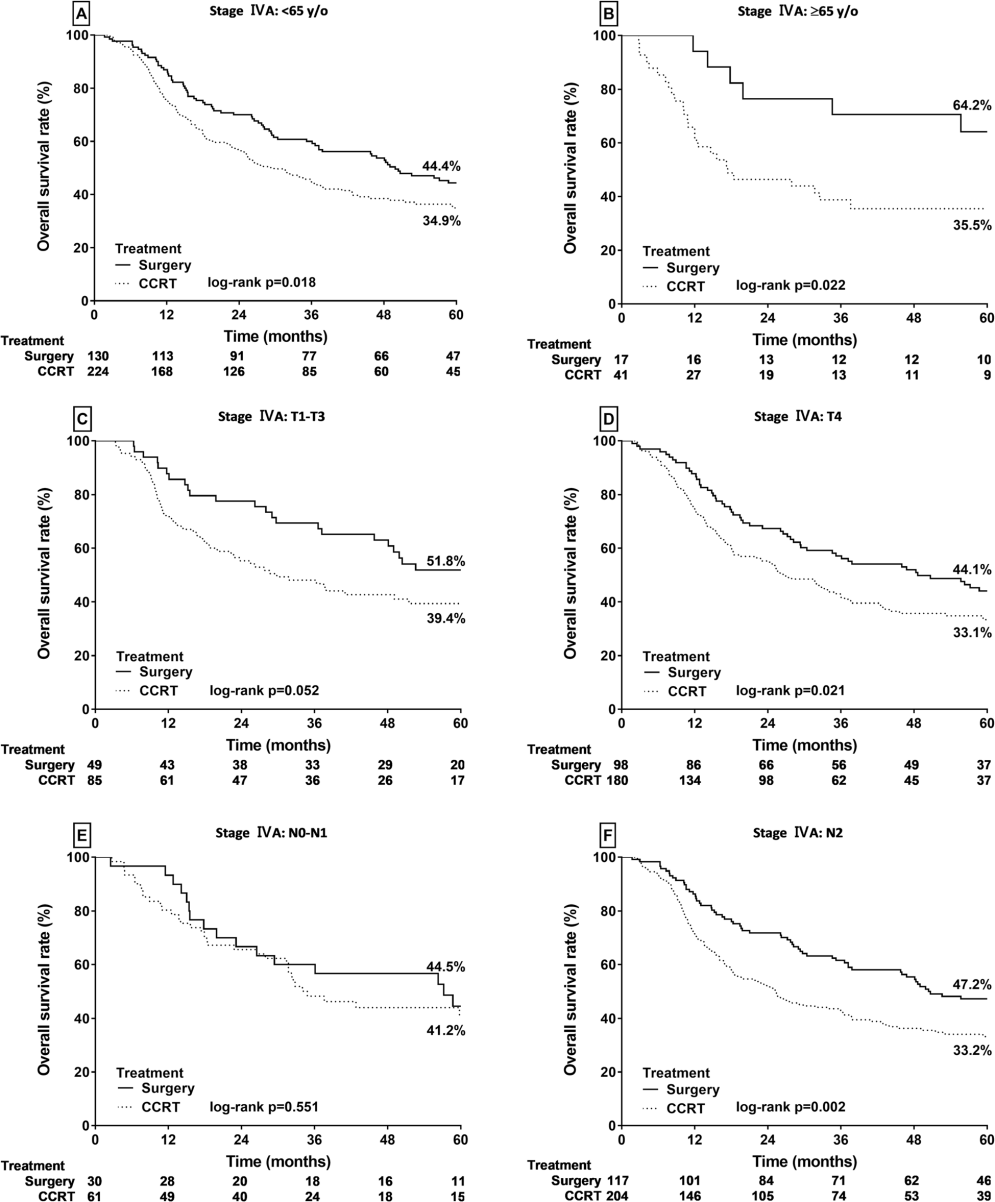
Kaplan–Meier analysis of OS rates in patients with stage IVA HPSCC. **a** Age < 65 years. **b** Age ≥ 65 years. **c** T1–T3 classification. **d** T4 classification. **e** N0–N1 classification. **f** N2 classification

To identify the subgroup of patients with stage IVA HPSCC that benefited most from PS treatment, we conducted subgroup analysis based on patient age (≥ 65 or < 65 years old), T and N classification, and treatment methods. As displayed in Fig. 4a and b, PS provided significantly higher OS rates than did CCRT treatment in patients aged < 65 (44.4% vs 34.9%, *p* = 0.018; Fig. 4a) and ≥ 65 (64.2% vs 35.5%, *p* = 0.022; Fig. 4b) years. Notably, the difference between the treatment method outcome was even more prominent in patients aged ≥ 65 years. After stratification for early (T1–T3) and advanced (T4) T classification of stage IVA HPSCC, PS led to higher OS rates than did definite CCRT in patients with T1–T3 (51.8% vs 39.4%, *p* = 0.052; Fig. 4c) and T4 (44.1% vs 33.1%, *p* = 0.021; Fig. 4d) classifications. Similarly, when we stratified stage IV patients by early (N0–N1) and advanced (N2) nodal disease, compared with definitive CCRT, PS led to significantly higher OS rates in patients with N2 classification (47.2% vs 33.2% for the CCRT group, *p* = 0.002; Fig. 4f); however, no significant difference appeared regarding OS in patients with N0–N1 classification (44.5% after PS vs 41.2% after CCRT, *p* = 0.551; Fig. 4e).

**Fig. 5:**
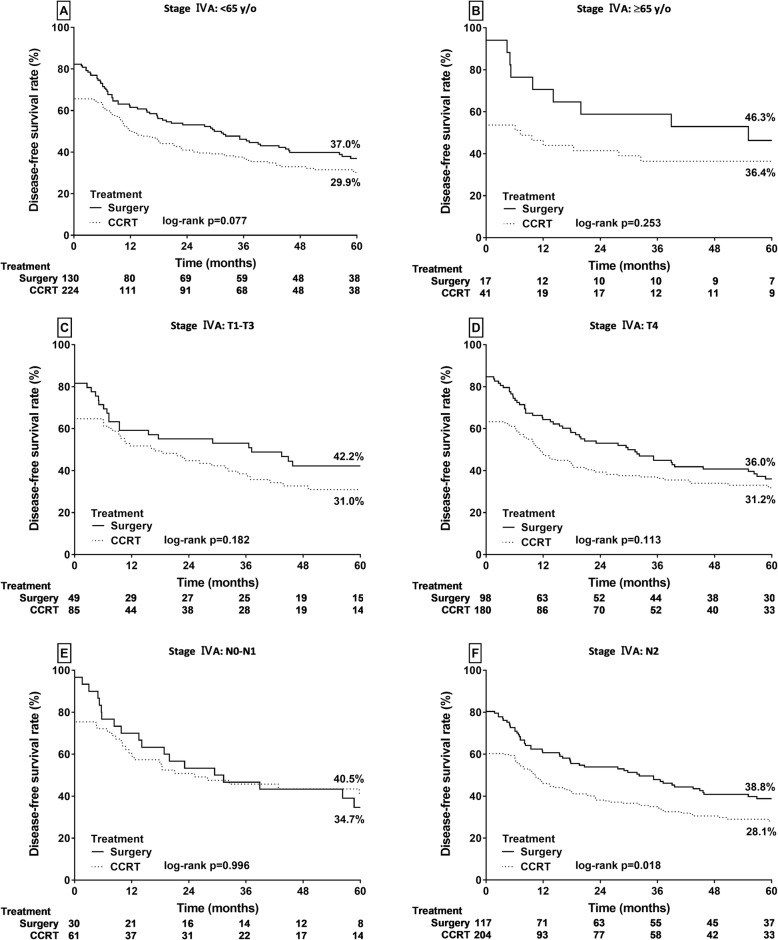
Kaplan–Meier analysis of DFS rates in patients with stage IVA HPSCC. **a** Age < 65 years. **b** Age ≥ 65 years old. **c** T1–T3 classification. **d** T4 classification. **e** N0–N1 classification. **f** N2 classification

PS also led to significantly higher DFS rates in patients with stage IVA HPSCC and N2 classification (38.8% vs 28.1% for the CCRT group, *p* = 0.018; Fig. 5f); however, no significant difference between the PS and CCRT groups in patients aged < 65 (37.0% vs 29.9%, *p* = 0.077; Fig. 5a) or ≥ 65 (46.3% vs 36.4%, *p* = 0.253; Fig. 5b) years or with T1–T3 (42.2% vs 31.0%, *p* = 0.182; Fig. 5c), T4 (36.0% vs 31.2%, *p* = 0.113; Fig. 5d), and N0–N1 (40.5% vs 34.7%, *p* = 0.99; Fig. 5e) classifications.

### Multivariate analysis of survival outcomes

Multivariate analysis revealed that the clinical stage and initial treatment modality (CCRT vs PS) were independent predictors of OS rates; moreover, advanced stages predicted low 5-year DFS rates (Table 2). In patients with advanced HPSCC, initial treatment with PS appeared to be associated with a better prognosis (Table 2): higher OS (HR 0.73; 95% CI 0.57–0.94, *p* = 0.014) and DFS (HR 0.83; 95% CI 0.66–1.04, *p* = 0.105) rates.

**Table 2:** Multivariate Cox proportional hazard model for overall survival and disease-free survival of advanced HPSCC patients

Variable	OS		DFS	
	HR (95% CI)	*p* value	HR (95% CI)	*p* value
Sex				
Female	Reference		Reference	
Male	1.74 (0.82–3.68)	0.150	1.39 (0.72–2.70)	0.332
Age (years)				
< 65	Reference		Reference	
≥ 65	1.01 (0.76–1.36)	0.929	0.97 (0.73–1.28)	0.965
Overall stages				
III	Reference		Reference	
IVA	1.67 (1.18–2.35)	0.004	1.64 (1.19–2.26)	0.057
IVB	2 98 (2.05–4.33)	< 0.001	3.01 (2.12–4.27)	< 0.001
Histology grade				
WD/MD	Reference		Reference	
PD/UD	0.99 (0.73–1.34)	0.944	0.97 (0.72–1.30)	0.826
Unclassified	1.06 (0.83–1.35)	0.632	1.10 (0.87–1.38)	0.440
Treatment				
CCRT	Reference		Reference	
Surgery	0.73 (0.57–0.94)	0.014	0.83 (0.66–1.04)	0.105

*OS* overall survival, *DFS* disease-free survival, WD well differentiated, *MD* moderately differentiated, *PD* poorly differentiated, *UD* undifferentiated, *CCRT* concurrent chemoradiotherapy

In the subgroup analysis of patients with stage IVA HPSCC, the multivariate Cox regression analysis revealed that PS was associated with higher OS (HR 0.69; 95% CI 0.51–0.93, *p* = 0.014; Table 3) and DFS (HR 0.78; 95% CI 0.59–1.03, *p* = 0.079) rates (Table 3); however, the difference was nonsignificant for the DFS rate. Of note, PS was strongly associated with a 67% lower mortality risk in stage IVA patients aged ≥ 65 years (HR 0.33; 95% CI 0.12–0.88, *p* = 0.027; Table 3). In addition, PS presented higher OS and DFS rates than did CCRT in patients with early (T1–T3) and advanced (T4) stage IVA HPSCC. However, the difference was nonsignificant, except for a marginal significance for OS in patients with T4 classification (HR 0.70; 95% CI 0.48–1.00, *p* = 0.053; Table 3). For stage IVA patients with N0–N1 classification, the difference in prognosis regarding OS and DFS rates between the two groups was nonsignificant (HR 0.92; 95% CI 0.46–1.86, *p* = 0.816 for OS; HR 1.11; 95% CI 0.51–2.15, *p* = 0.762 for DFS; Table 3). Nevertheless, in stage IVA patients with N2 classification, PS was associated with significantly lower mortality risks than was CCRT, based on the OS (HR 0.64; 95% CI 0.46–0.90, *p* = 0.01; Table 3) and DFS (HR 0.71; 95% CI 0.52–0.97, *p* = 0.033) rates.

**Table 3:** CCRT versus primary radical surgery in subgroup analysis by age and T and N classification following multivariate analysis of stage IVA HPSCC patients

Variable	OS		DFS	
	HR (95% CI)*	*p* value	HR (95% CI)*	*p* value
Treatment				
CCRT	Reference		Reference	
Surgery	0.69 (0.51–0.93)	0.014	0.78 (0.59–1.03)	0.079
Subgroup				
Age < 65 years				
Treatment				
CCRT	Reference		Reference	
Surgery	0.76 (0.55–1.04)	0.087	0.82 (0.61–1.10)	0.188
Age ≥ 65 years				
Treatment				
CCRT	Reference		Reference	
Surgery	0.33 (0.12–0.88)	0.027	0.53 (0.22–1.26)	0.149
T1–T3				
Treatment				
CCRT	Reference		Reference	
Surgery	0.67 (0.39–1.15)	0.143	0.76 (0.46–1.24)	0.267
T4				
Treatment				
CCRT	Reference		Reference	
Surgery	0.70 (0.48–1.00)	0.053	0.79 (0.56–1.12)	0.186
N0–N1				
Treatment				
CCRT	Reference		Reference	
Surgery	0.92 (0.46–1.86)	0.816	1.11 (0.51–2.15)	0.762
N2				
Treatment				
CCRT	Reference		Reference	
Surgery	0.64 (0.46–0.90)	0.010	0.71 (0.52–0.97)	0.033

*OS* overall survival, *DFS* disease-free survival, *WD* well differentiated, *MD* moderately differentiated, *PD* poorly differentiated, *UD* undifferentiated, *CCRT* concurrent chemoradiotherapy

*Model adjusted for sex, age, and histology grade

## Discussion

In this multicenter retrospective study, we investigated the survival outcomes of patients with advanced-stage HPSCC who received either definitive CCRT or radical PS followed by adjuvant therapy, both of which are considered feasible options for advanced HPSCC treatment. The results revealed a significant survival advantage from PS for both OS and DFS rates in advanced HPSCC treatment. The multivariate analysis results confirmed the survival advantage of PS over CCRT, based on the 27% risk reduction noted in the overall mortality—partially explained by the diminished tumor volume leading to potentially higher local control rates [7, 18, 19]. Subgroup analysis based on clinical stage indicated that in patients with stage IVA HPSCC, PS has higher OS and DFS rates than does definitive CCRT—whereby the mortality risk is reduced by 31%.

Furthermore, for stage IVA patients with N2 classification, PS reduced the mortality risk by nearly 30% for both OS and DFS rates—consistent with the results of two studies that noted that the neck nodal metastatic burden significantly affected the survival outcomes and the organ preservation rate [6, 20]. In patients with stage IVA HPSCC aged ≥ 65 years, PS considerably reduced overall mortality risk by 67%. These results suggested that PS with or without adjuvant therapy may provide improved survival than does definitive CCRT for the treatment of advanced-stage HPSCC, particularly in patients with stage IVA.

The current results were derived from a relatively large population of patients with advanced HPSCC from four hospitals with a wide variety of clinicopathological characteristics regarding tumor histology, cancer staging, and primary treatment based on the preferences of various physicians. The current results thus provide a cross-sectional view for the management of advanced HPSCC cases during the study period and afford a fair comparison of the survival outcomes between the two major treatment modalities. The current study revealed that in patients with stage III HPSCC with medical disadvantages regarding surgery or who initially refused to undergo surgery, CCRT remained a reasonable and feasible alternative treatment choice with regard to survival outcomes.

PS and CCRT treatment for advanced HPSCC resulted in substantial complications and sequelae and required detailed multidisciplinary consultations with the patients. First, the results of organ preservation treatment for patients with advanced HPSCC are significantly inferior to the results reported of laryngeal preservation protocols of laryngeal cancer; furthermore, treatment toxicity is common and typically severe [12, 21]. Definitive CCRT can be accompanied by major complications in patients with T4a classification [22], such as high feeding tube placement rates [23], high multiple surgical intervention prevalence [16], and serious intractable complications during salvage surgery, such as poor wound healing and pharyngocutaneous fistulae [24]. Consequently, organ preservation therapy, such as CCRT in the current study, for advanced HPSCC may preserve a dysfunctional organ but worsen the survival based on the current study results. Because PS can provide a higher local control rate, fewer complications, and better survival outcomes than can definitive CCRT (according to the results of a previous study and the current study), PS may be the optimal treatment in patients with stage IVA HPSCC, particularly in patients older than 65 years with or without N2 nodal spread [19].

Primary tumor and metastatic nodal volumes can be crucial prognostic predictors when treating advanced HPSCC. Tsou et al. reviewed 51 patients with advanced HPSCC and proposed that primary tumor volume was associated with the local control rate and was the most critical factor when considering CCRT as the initial treatment method [20]. In another retrospective analysis, Anna et al*.* reviewed 78 patients who underwent definitive CCRT for stages III–IV HPSCC and discovered that primary tumor volume was a better prognostic factor than T or N classification [25]. A primary tumor volume greater than 40 mL was reported to be a substantial indicator in patients with HPSCC treated through surgery [26]. Axon et al. analyzed 143 patients with postcricoid carcinoma and noted that the presence of metastatic nodal disease at presentation was the most significant prognostic factor [6]. Tsou et al. also analyzed patients with HPSCC in a series of studies and discovered that metastatic nodal volume, nodal counts, and nodal levels were significant factors that affected survival outcome and the organ preservation rates in patients with HPSCC treated by CCRT [7, 20]. The results of these studies have suggested that PS followed by adjuvant treatment minimized the tumor and nodal burden considerably; therefore, PS results in a better survival advantage than does CCRT in patients with advanced HPSCC.

In patients with stage IVB HPSCC with either extensive primary tumors (T4b) or a large nodal burden (N3), whether PS followed by adjuvant therapy provides better survival than definitive CCRT remains unclear. Because most cases of stage IVB HPSCC were considered unresectable at the time of diagnosis, only 14.8% of patients with stage IVB received PS treatment as the initial intervention in the present study, and the Kaplan–Meier survival analysis revealed no statistically significant difference between initial PS and definitive CCRT treatment. Studies have reported the effectiveness of organ preservation treatments in advanced HPSCC, including at stage IVB [10, 27]. Slotman et al. reviewed 54 patients with advanced HPSCC in the pyriform sinus and discovered that the regional control rate was significantly lower in patients with N3 classification than in those with N0–N2 despite a higher radical radiotherapy dose [5]. In patients diagnosed as having clinical T4b HPSCC, the tumor invaded the prevertebral fascia and could not be well differentiated from non-neoplastic changes in the prevertebral space through magnetic resonance imaging or computed tomography [28]. Therefore, the proportion of patients with clinical T4b may be overestimated from imaging studies, and the nonsurgical modality was the typical treatment used in most of these scenarios. Future studies enrolling larger cohorts are needed to elucidate the best treatment strategy for patients with stage IVB HPSCC.

## Conclusions

Despite current multidisciplinary treatments, HPSCC remains a detrimental disease with a poor prognosis among the major head and neck malignancies. In this multi-institutional review of the survival outcomes of four hospitals, the current study revealed that primary treatment with PS provided higher OS and DFS rates in patients with stage IVA HPSCC, particularly among those with N2 classification and ages ≥ 65 years. In patients with stage III or IVB HPSCC, CCRT remained a reasonable and feasible treatment modality in survival outcomes and organ preservation. Further investigation to elucidate most appropriate personalized treatment for these patients is warranted.

## Data Availability

The datasets used and/or analyzed during the current study are available from the corresponding author upon reasonable request.

## Abbreviations

OS: Overall survival
DFS: Disease-free survival
HPSCC: Hypopharyngeal squamous cell carcinoma
CCRT: Concurrent chemoradiotherapy
WD: Well-differentiated squamous cell carcinoma
MD: Moderately differentiated squamous cell carcinoma
PS: Primary surgery
PD: Poorly differentiated squamous cell carcinoma
UD: Undifferentiated carcinoma
HR: Hazard ratio
Cls: Confidence intervals
